# Deep learning-based transcriptome data classification for drug-target interaction prediction

**DOI:** 10.1186/s12864-018-5031-0

**Published:** 2018-09-24

**Authors:** Lingwei Xie, Song He, Xinyu Song, Xiaochen Bo, Zhongnan Zhang

**Affiliations:** 1 Xiamen University, Xiamen, 361005 China; 2 Beijing Institute of Radiation Medicine, Beijing, 100850 China

**Keywords:** Drug-target interaction, Deep learning, LINCS project, Transcriptome data

## Abstract

**Background:**

The ability to predict the interaction of drugs with target proteins is essential to research and development of drug. However, the traditional experimental paradigm is costly, and previous *in silico* prediction paradigms have been impeded by the wide range of data platforms and data scarcity.

**Results:**

In this paper, we modeled the prediction of drug-target interactions as a binary classification task. Using transcriptome data from the L1000 database of the LINCS project, we developed a framework based on a deep-learning algorithm to predict potential drug target interactions. Once fully trained, the model achieved over 98% training accuracy. The results of our research demonstrated that our framework could discover more reliable DTIs than found by other methods. This conclusion was validated further across platforms with a high percentage of overlapping interactions.

**Conclusions:**

Our model’s capacity of integrating transcriptome data from drugs and genes strongly suggests the strength of its potential for DTI prediction, thereby improving the drug discovery process.

## Background

The identification of drug-target interactions (DTIs) is significant to drug research and development (R&D). The ability to predict DTIs has been applied widely for drug repositioning and for anticipating adverse reactions [1, 2]. Large numbers of DTIs have been uncovered in databases such as DrugBank, Matador, and CTD, but many DTIs remain to be discovered [3–5]. Although high-throughput screening technology is available, the traditional strategy used for discovering new DTIs is still time consuming and costly.

Researchers have developed a variety of computational algorithms to facilitate the prediction of DTIs. For example, Campillos et al. proposed an algorithm to predict whether two drugs share target protein based on similarities of phenotypic side-effect [6]. Bleakley et al. developed a supervised method, shown as a bipartite graph, to predict drugs targeting a given protein [7]. The AUC of the bipartite local model method in different datasets vary from 74.5 to 97.3%. Wang et al. introduced the framework of restricted Boltzmann machines to predict DTIs with a high AUC [8]. Yamanishi et al. predicted DTIs by integrating chemical and genomic spaces into a bipartite graph [9]. The AUC of the bipartite graph learning method vary from 84.3 to 90.4%.

In principle, both data source and prediction algorithm contribute to the performance of DTI prediction. Researchers have attempted to use various aspects of drug informatics data, such as cellular response data, pharmacological data, chemical data, and side effect data, to identify novel DTIs [6, 10, 11]. However, the performance of *in silico* prediction has been held back by the wide variety of data production platforms and scarcity of data.

To address the problem of data scarcity, the National Institute of Health (NIH) launched the pilot phase of the Library of Integrated Network-based Cellular Signatures (LINCS) project in 2010. This project aims to provide a comprehensive map of multilevel cellular responses when cells are exposed to various perturbations, including small molecule compound stimulation and gene knockdown (http://www.lincsproject.org/LINCS). The L1000 database of the LINCS project includes millions of genome-wide expression profiles gathered from cell lines stimulated by more than 20,000 small molecule compounds, or when more than 4000 genes were knocked out in the respective cell lines. The L1000 database provides a unified and extensive gene expression profile source for DTI prediction.

In this study, supported by increased availability of GPU computing and expanded data sources, we explored the possibility of deep learning method to discovery new DTIs based on transcriptome data from drug perturbation and gene knockout trials in the L1000 database. Inspired by the intrinsic nonlinear patterns revealed by the LINCS project, we proposed a framework that offers better prospects for inferencing and for DTI prediction [12]. First, we developed a permutation of gene expression data of drugs and genes, both from the L1000 database, in a serial manner according to known DTIs in the DrugBank database [3]. Second, the input space, which consisted of all positive samples and distributed negative samples, is for training and evaluating our proposed deep neural network (DNN) model that had only 2000 hidden units. After forward propagation, the feature dimensionality was reduced approximately 200 times. By the conclusion of training, the DNN model derived a decision boundary to classify positive and negative samples with the desired accuracy, and the model was able to predict reliable DTIs. Last, we analyzed the predicted results further using a distance metric (D-score) and cross-platform comparison. Further research proved that our framework could predict a certain number of novel DTIs that were validated by known experiments in other databases, including CTD, DGIdb, and STITCH [5, 13, 14]. The experimental results showed that our DNN model is capable of extracting low dimensional features representation and can classify samples accurately. Furthermore, our framework can integrate transcriptome data from drugs and genes, indicating the strength of its potential for DTI prediction, thereby improving the drug discovery process.

## Methods

In this section, we discuss methods for discovering new DTIs, including the use of data from the L1000 and DrugBank databases, the problem definition, and our approach to deep learning.

To address the challenge of predicting unknown DTIs, we modeled the problem as a binary classification task. Firstly, for the original dataset, we selected a large number of expression data from various drug perturbation and gene knockout trails. Some of genes were target proteins while others were not. However, the number of negative data was far greater than the positive data in PC3 cell line. The whole input space contained all positive data, along with uniformly sampled expression data from the negative sample space. Then we described the feature space based on combinations of the expressions from drugs and genes. Last, after fitting training achieved highly accurate data, we used the model for DTI prediction.

### Data from the L1000 database

The LINCS project hopes to decipher how cells respond to various genetic and chemical stresses. By the time of completion, the pilot phase of the project had generated more than 660,000 gene expression profiles from perturbation of more than 10,000 small molecule compounds, and had gathered more than 440,000 gene expression profiles of more than 4000 genes with knockout mutations.

The L1000 database provides direct measurement of the expression profiles of only 978 landmark genes, and uses correlations to these genes to infer the remaining ∼20,000 gene expressions. The data structure of the LINCS project, like the TCGA project (https://cancergenome.nih.gov/), consists of four levels. Level 1 data represents the expression value of the 978 landmark genes. Level 2 data represents the normalized expression value of the 978 landmark genes. Level 3 data records genome-wide expressions. Level 4 data records the Z-score of genome-wide gene expressions, which is used in this research for drug perturbations and gene knockdown perturbations in the PC3 cell line.

Since the LINCS project is still on the way, few perturbation’s name can map to the drugs from the DrugBank database. We selected the Level 4 data of 480 FDA-approved drug perturbations and 4363 gene knockout perturbations in the PC3 cell line. We used the landmark genes’ Z-score to reduce the feature dimensionality.

Firstly, we computed the Pearson correlation coefficient matrix for trails of a certain drug or a gene. Next, we used the *k*-means algorithm to divide the drugs or genes into several clusters. If the number of drug samples is more than 2 but less than 5, we set *k* as 2. If it is more than 5 and less than 15, we set *k* as 3. And if it is over 15, we set *k* as 4. And we chose the cluster with the maximum intra-class Pearson correlation coefficient as the representation of the drug or gene, denoted by *S*_1_. Meanwhile, to retain more of the information about the trials of the drug or gene, we averaged all trial data as an independent sample *S*_2_. Last, we constructed a credible set *S* of the drug or gene using *S*_1_ and *S*_2_. The features of drugs in *S* are the 978 landmark genes.

### DTI database

In this paper, we used the DTIs in DrugBank database version 5.0, a comprehensive drug informatics data source that records chemical, pharmacological, and pharmaceutical features of more than 8000 drugs, to train and evaluate our model [3]. To compare cross-platform data, we used the PubChem ID as the identifier of drugs across the L1000 and DrugBank database. Finally, we filtered 918 DTIs from 415 drugs and 350 targets in the DrugBank database to use as the gold standard.

In addition, to validate the DTIs predicted by our model from 623 drugs and 378 targets, we used three datasets derived from CTD, DGIdb, and STITCH. For the 623 drugs, we selected 140,972 interactions from CTD, 19,654 interactions from DGIdb, and 958 interactions from STITCH.

### Problem definition

For our research, the transcriptional response data of drugs and target proteins perturbation were taken from L1000 database, and the DrugBank database provided the relationships between them. To explore new DTIs, the DTI prediction was modeled as a binary classification task, and the proposed approach took two data channels (drug channel and gene channel) as input. Each sample was constructed by fusing a drug datum and a gene datum. The definition details are as follows.

#### Definition 1.

Drug matrix *DM* is an *m* by *n* matrix that consists of all drugs, which is the drug perturbation profile in the dataset *S*. *m* is the number of drugs, and *n* is the number of landmark genes. Each line means one drug.

#### Definition 2.

Gene matrix *GM* is an *q* by *n* matrix that consists of all genes, which is the gene knockdown perturbation profile in the dataset *S*. *q* is the number of genes, and *n* is the number of landmark genes. Each line means one gene.

#### Definition 3.

Features *DM*_*i*,*j*_ and *GM*_*i*,*j*_ are both real numbers; each corresponds to the expression of the *j*th landmark gene for sample *i*.

#### Definition 4.

Label matrix *LM* is a *q* by *m* matrix. *LM*_*i*,*j*_ is the single label for the interaction between gene *i* and drug *j*. If *LM*_*i*,*j*_ = 0, then the combination of gene *i* and drug *j* is either an unlabeled sample or a negative sample, depending whether gene *i* is one of the target proteins or not. Otherwise, gene *i* (also suggest target protein *i*) is a target of drug *j* recorded in the DrugBank database, and the combination of gene *i* and drug *j* is a positive sample.

#### Definition 5.

Classification matrix *CM* is an *l* by *k* matrix. *l* = *mq* is the number of all possible DTIs between *m* drugs and *q* targets, and *k* = 2 indicates the positive or negative interaction between each drug-target pairs. *CM*_*i*,0_ is the probability of sample *i* (the *i*th DTI) belonging to the negative class. *CM*_*i*,1_ is the probability of sample *i* belonging to the positive class.

### Supervised learning

In supervised learning, hypothesis space *F* is the set of joint probability distributions and conditional probability distributions. If the model *f* is selected as a decision function, for any input *X* the predicted value Y*=*f*(*X*) is obtained. The objective function *L*(*Y*, *f*(*X*)), a real-valued function of *f*(*X*) and *Y*, is constructed for measuring the nearness between predicted values and true values. Since the loss value becomes smaller, the fit of the model improves on the training sets.

As a result of the improved availability of data for cell biology and drug chemistry, our proposed DNN was able to serve as an powerful tool for DTI prediction. The computational power was derived in two ways: first, through a massively parallel distributed structure, and second, through the network’s ability to learn and generalize. The DNN had a built-in ability to adapt parameters according to the changes of the surrounding environment.

Every neuron in the DNN was nonlinear. This property was highly significant, particularly where the underlying physical mechanism responsible for generation of the input signal was inherently nonlinear, and potentially could be affected by the global activity of all other neurons in neural network. Above all, the DNN automatically extracted more abstract features representing raw chemical and biological data. Nonetheless, the architecture remains a huge challenge when feature space is high-dimensional.

In this study, DTI prediction was modeled as a binary classification task in machine learning domain. Therefore, the input layer contained two channels for stacking drug data and gene data from the L1000 database as input. There were two neurons in output layer for binary classification that indicated the effectiveness of the drug relative to the gene. The performance of the network was impacted by the depth and breadth of the layers. If the architecture was too complicated, the risk of over-fitting increased; otherwise, the performance declined. The optimal number of hidden layers and neurons, the dropout rejection rate, and the class imbalance weight were investigated on *K*-fold cross-validation. Last, a Softmax regression, as defined in (1), was adopted for output layer.

1 $$\begin{array}{@{}rcl@{}} f_{\theta}\left(x_{i}\right) &=& \left[\begin{array}{c}p(y_{i} = 1 | x_{i}; \theta_{1}) \\ p\left(y_{i} = 2 | x_{i}; \theta_{2}\right) \\\vdots \\ p\left(y_{i} = k | x_{i}; \theta_{k}\right)\end{array}\right] \\ &=&\frac{1}{ \sum_{j=1}^{k}{e^{\theta_{j}^{T} x_{i} }} } \left[\begin{array}{c}e^{\theta_{1}^{T} x_{i}} \\ e^{\theta_{2}^{T} x_{i}} \\ \vdots \\ e^{\theta_{k}^{T} x_{i}}\end{array}\right] \end{array} $$

**Fig. 1:**
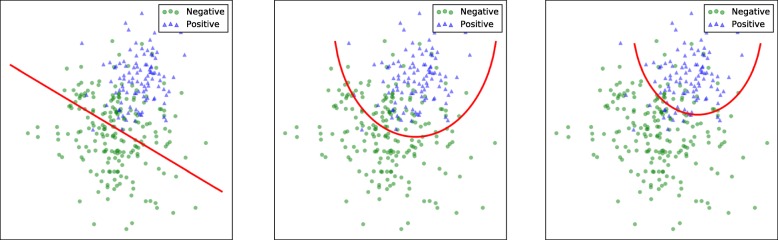
The decision boundary of the DNN. The DNN fit training data with a nonlinear decision boundary rather than a hyperplane in high dimensional space. The final decision boundary approximated positive clusters iteratively during training procedure, even though sacrificing a little of validation accuracy

During the training procedure, each layer was randomly initialized first. Then each neuron was activated by ReLU with strong biological stimulation and mathematical justification. To ensure that a trained model would have better potential for DTI prediction, training was completed by AdamOptimizer to minimize the cross entropy cost function with L1 penalty for the probabilty of negative samples belonging to negative class, as defined in (2). After training, the model had better potential for DTI prediction even though the ratio sacrificed a small amount of accuracy. As shown in Fig. 1, the DNN fit the training data with a nonlinear decision boundary (middle plot) instead of a hyperplane (left plot). Moreover, the ratio of positive (3826) to negative (7652) samples provided more information that made the network learn the features of negative samples. The rebuilt objective function paid more attention to real negative class in order to push the decision boundary closer to the center of the positive class cluster (right plot).

2 $$ \begin{aligned} {} (\theta,{b}) &\,=\, \arg\min_{\theta,{b}}\frac{1}{m}\! \left[ \sum\limits_{i=1}^{m} -y_{i} \ln f_{\theta}\left(x_{i}\right) \right. \\ &\quad\,-\, \left. \left(1\,-\,y_{i}\right) \!\ln\! \left(1\,-\,f_{\theta}\left(x_{i}\right)\right) \,-\, \eta\sum_{{x_{i}} \in Negative}\!\left\|{CM}_{i,0}\!\right\|_{1}\! \right] \end{aligned}  $$

We compared the trained model with other methods by using the F-score, validation accuracy, and predictive error (as defined in (3)) at each sample *x*_*i*_. Every interaction prediction was measured by *CM*_*x*,1_, and further analyzed through the distance from the unlabeled sample point to the decision boundary, as defined in (4). This distance function was inspired by prior research that converted the distance function to probability in a tree kernel-based SVM [15], and the hypothesis of Softmax was equivalent to SVM for binary classification.

3 $$ \text{PE}\left({x_{i}}\right) = {CM_{x_{i},y_{i}}^{\text{other}}} - {CM_{x_{i},y_{i}}^{\text{DNN}}}  $$

4 $$ \text{D - score}({f}\, |{X}) = \ln \left(\frac{{CM_{X,1}}}{ 1 - {CM_{X,1}} }\right)  $$

## Results

As discussed earlier in this paper, we modeled the discovery of new DTIs as a binary classification task. The whole dataset contained all the expression data of drugs, target and non-target protein genes. However, there were more negative samples (combining drug data and non-target protein gene data) than the number of positivie samples (combining drug data and target protein gene data). Therefore, the input space consisted of all positive samples and uniformly sampled negative samples. As the result of some intrinsic linear and nonlinear patterns in the LINCS project [12], linear regression (LR) was adopted to capture linear features [16], but some nonlinear features were inevitably ignored. However, others, e.g., Random Forest (RF) [17], were responsible for extracting nonlinear features for classification. All models were implemented in PC3 cell line with the same promising ratio of positive to negative.

### Deep learning results

DNNs are multilayer systems of connected and interacting artificial neurons that perform various data transformations. They have several hidden layers of neurons, which allows for adjustment of the data abstraction level. The ability to learn at the higher abstraction level makes DNNs an effective and promising tool for working with chemical and biological data. In the LINCS project, linear features can be captured by linear methods, but classification performance reaches a plateau because such methods ignore complex nonlinear relationships between the expressions of genes.

**Fig. 2:**
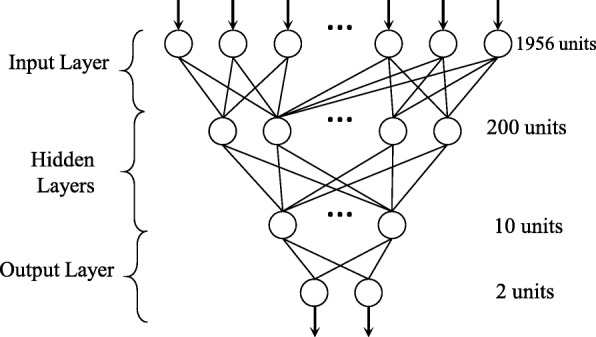
The architecture of DNN. The network included one input layer with 1956 neurons, two hidden layers with 200 neurons and 10 neurons respectively, and one Softmax layer as the binary classification layer

In order to learn hierarchical nonlinear features systematically, we designed a DNN that included one input layer with 1956 neurons corresponding to the dimensionality of features, two hidden layers with 200 neurons and 10 neurons respectively, and one Softmax layer as the binary classification layer. After feature extraction, each sample was represented as a 10-dimensional feature vector, and such feature learning contributed effectively to classification. However, overfitting is a serious problem for fully connected network, and a complicated network is time consuming when using forward propagation. This is why we adopted the dropout technique to simplify network architecture. The critical idea is to drop neurons randomly during training to prevent these neurons from co-adapting too much [18]. The architecture of DNN as shown in Fig. 2.

The trained model was used for exploring unknown DTIs. The ratio of positive to negative samples was selected as 1:2, and the objective function was rebuilt by weighting the sum of *CM*_*i*,0_ of all negative samples, as defined in (2). These steps made the network have better potential for DTI prediction.

**Fig. 3:**
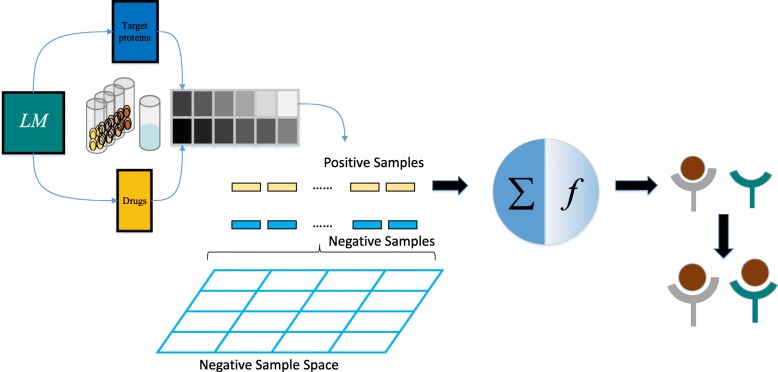
The whole pipeline. The complete process flow of the framework contained feature fusion, negative data sampling, model training, and prediction

As shown in Fig. 3, the process flow of the framework as a whole contained feature fusion, negative data sampling, model training, and DTI prediction. In the PC3 cell line, too many negative samples introduce serious class imbalance problem. Therefore, the negative samples were selected at uniform intervals from the whole negative sample space. To model the relationships between drugs and genes, the two data channels were used as a combination instead of a separation. Thus, each sample was created by fusion of a drug datum and a gene datum at the feature level without any drops before feeding into any model. We used this procedure because the original features that were sufficient statistics fully contained information of original data at the feature level. Although we tried direct methods to put drug data and gene data together through simple operations (e.g., addition, subtraction, multiplication, and division), these effort did not generate additional redundant features or a more complex feature space. However, such operations were irreversible and changed the expressions of several key loci, resulting in information that contributed to classification loss.

**Fig. 4:**
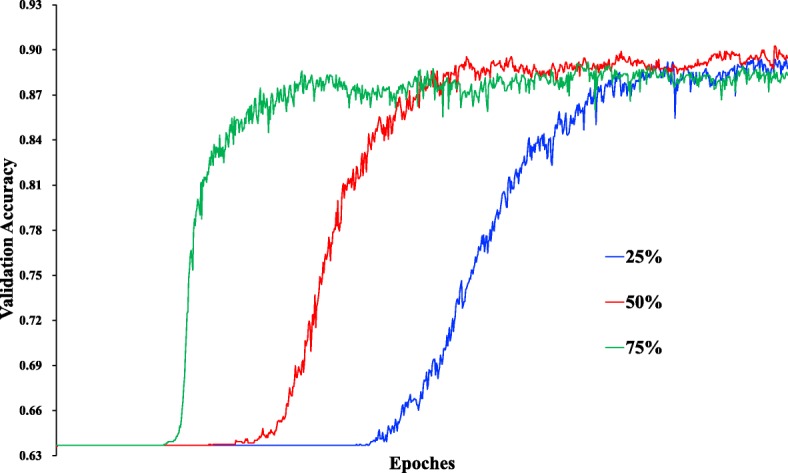
The validation accuracy under different dropout rates. When the dropout rate equaled 50%, the performance of the DNN was the best, because the trained model was assembled by sub-models, and the number of possible sub-models is 2 ^*t*^, where *t* denotes the number of hidden neurons in second hidden layer

**Fig. 5:**
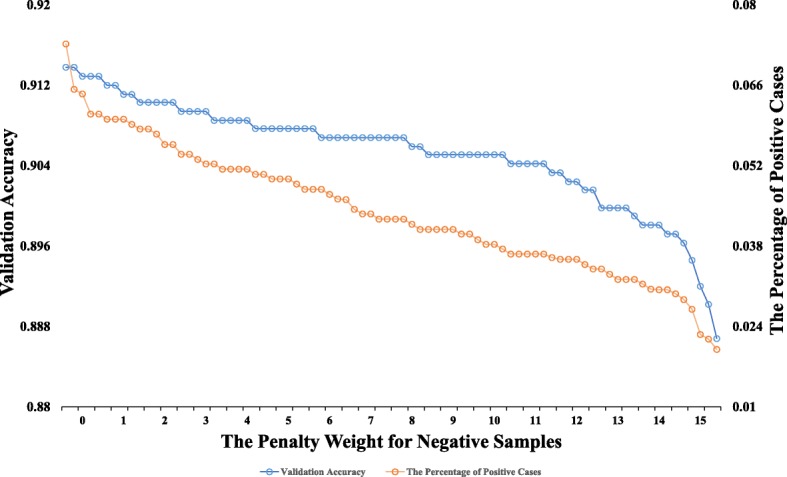
The experimental results of different penalty weights for negative samples. In order to keep the trade-off between validation accuracy and the percentage of positive cases, the penalty weight for negative samples was selected as 10

**Fig. 6:**
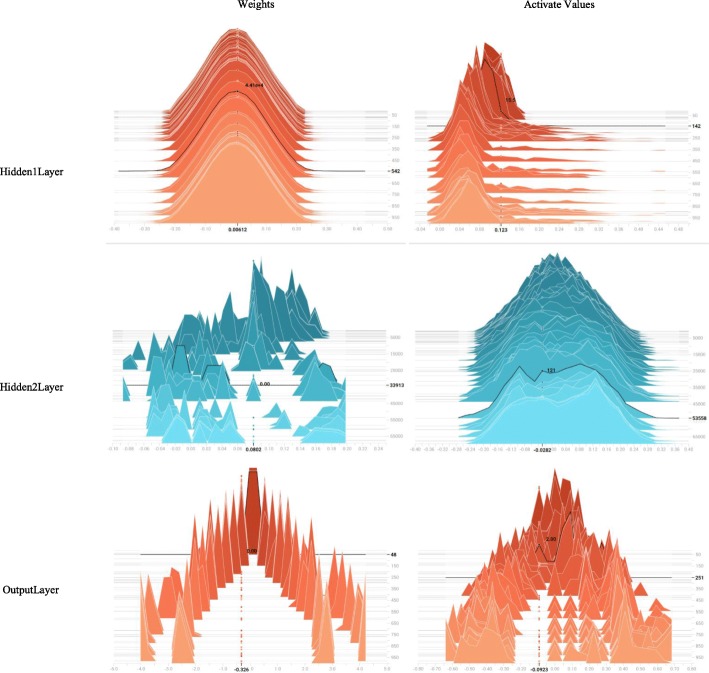
The distribution for weights and activate values coming off particular layers. These histograms show weights and activation values varied over time. The x-axis presents real value and the y-axis presents training steps

For the original feature combination from two data channels, we constructed an expression map for each sample in a serial manner. This method preserved all original information, and did not introduce more redundant noises. In the training procedure, the probability of the dropout rate was selected as 50% (as shown in Fig. 4). This selection meant that the final model integrated 2 ^*t*^ sub-models, where *t* denotes the number of hidden neurons in the second hidden layer. The weight *η* in the objective function was selected as 10 based on observations of the learning curve (as shown in Fig. 5). The AdamOptimizer functioned as an objective function optimizer with a learning rate of 1e−4 to train the DNN. The distribution of values in particular layers over time is shown in Fig. 6. The trained model fit training data with over 98% train accuracy, and generalized validation data with approximately 90% validation accuracy.

### Ablation study

In this paper, different types of methods were introduced for the ablation study, including RF, LR, Voting Classifier (VC), and Gradient Boosting Decision Tree (GBDT) using the L1000 dataset. We compared the performance of our proposed DNN with other methods by examining the F-score, validation accuracy, percentage of positive cases (PoPC), and predictive error. LR is responsible for linear analysis because it can capture effective linear features. RF and VC, as ensemble classifiers consisting of multiple weak classifiers, are adopted widely in classification tasks. GBDT showed excellent performance in recommender systems because it has the advantage of combining different features.

**Fig. 7:**
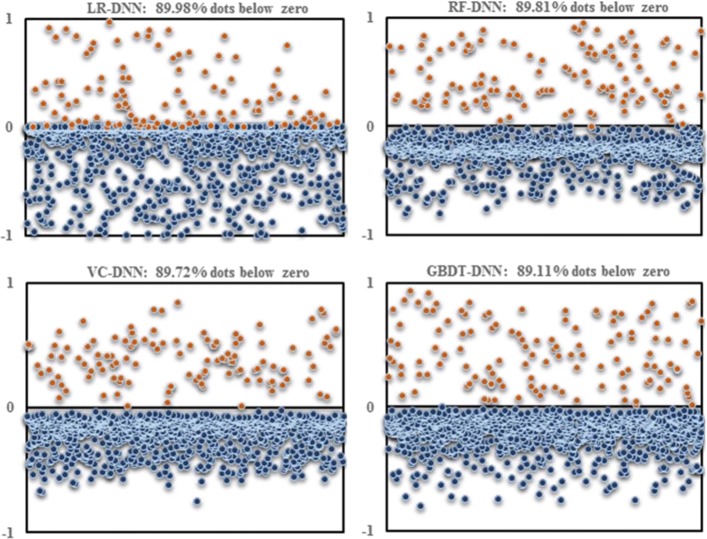
The predictive errors between other models and the DNN. Each dot represents the difference between the DNN and other models. If the dot had a negative label, the predictive error was from the *CM*_*i*,0_ of other models minus the *CM*_*i*,0_ of the DNN. Otherwise, the predictive error was from the *CM*_*i*,1_ of other models minus the *CM*_*i*,1_ of the DNN. Therefore, if more dots were below the horizontal line, the performance of the DNN was better than the performance of other models. Otherwise, the DNN performance was worse

As shown in Table 1, the F-score and validation accuracy of our DNN were better than other methods, and the PoPC on totally 1032658 unlabeled data was at least six times less. Such reliable PoPC that is premised on accurate fitting benefit from the imbalanced ratio of positive to negative samples. In addition, if the predictive error of *x*_*i*_, as defined in (3), was less than 0, the performance of the model was worse than the performance of the DNN. Otherwise, the other models were shown to be better than the DNN. As shown in Fig. 7, more than 89% of the dots were below the 0 (horizontal) line in the PC3 cell line. In other words, the results suggested that the performance of the DNN that was designed by us was much better than other classic classification models.

**Table 1:** Performance comparisons across methods

	Validation accuracy	F-score	PoPC
LR	76.84% ±0.96	68.88% ±0.05	38.11% ±2.85
RF	87.12% ±1.50	77.57% ±4.97	23.40% ±1.77
VC	90.00% ±0.05	84.45% ±0.71	29.85% ±3.01
GBDT	90.46% ±0.02	85.86% ±1.70	28.41% ±2.01
DNN	90.53% ±1.44	86.38% ±1.96	03.98% ±1.10

### Validation and analysis of novel predictions

**Fig. 8:**
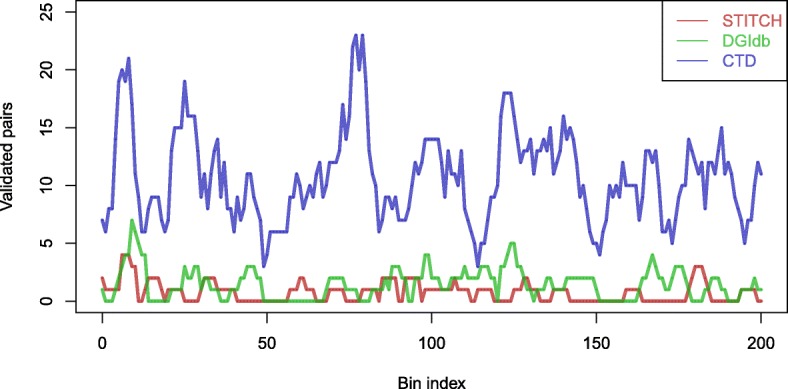
The overlap curves between predicted interactions and known DTIs. We computed the difference in the number of overlapping DTIs between the predicted results and the DTIs from the three databases. Then, we count the number of overlapping DTIs in the sliding bins of 500 consecutive DTIs

After training and evaluating our model, we utilized it to predict novel DTIs. To validate whether our prediction results were in accord with current knowledge, we examined the predicted DTIs using other DTI database, including STITCH, DGIdb, and CTD. A total of 221 pairs were found in STITCH, 466 pairs in DGIdb, and 3254 pairs in CTD. After that, we used D-score to rank all predicted DTIs, and calculated pairs count that overlap between the predicted results and the interactions from the other three databases. Then we counted the number of overlapping pairs in the sliding bins of 500 consecutive interactions (as shown in Fig. 8). It suggests that our model can predict novel DTIs validated by known experiments in other databases.

**Fig. 9:**
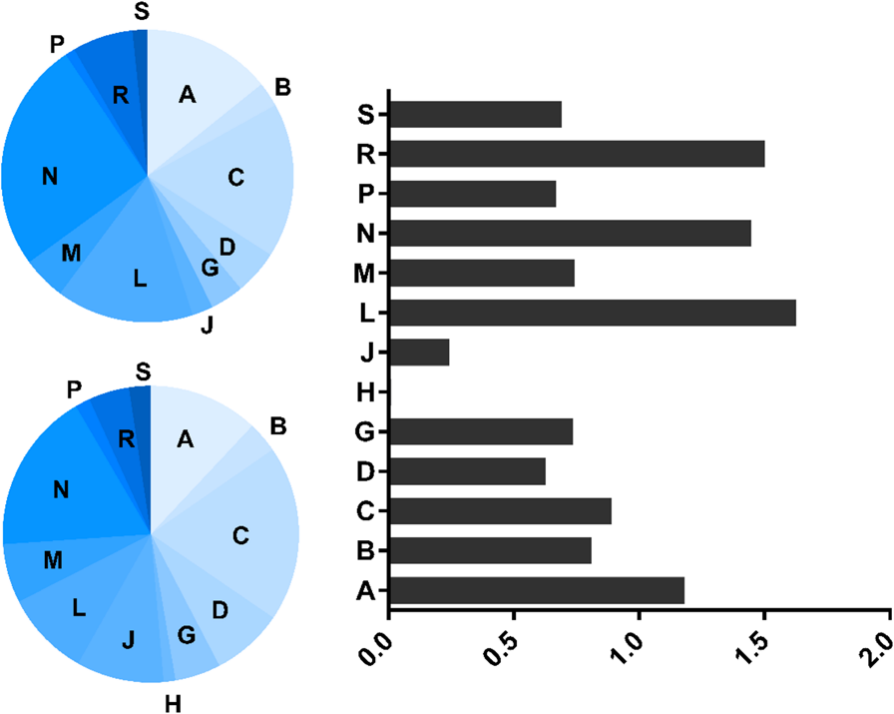
Distribution of ATC labels between DTIs in the known (left-top panel) and predicted (left-bottom panel) interactions. The relative ratio between predicted and known DTIs for each ATC label is shown in the right panel. ATC labels include the following. A: alimentary tract and metabolism; B: blood and blood-forming organs; C: cardiovascular system; D: dermatologicals; G: genito-urinary system and sex hormones; H: systemic hormonal preparations, excluding sex hormones and insulins; J: anti-infectives for systemic use; L: anti-neoplastic and immunomodulating agents; M: musculoskeletal system; N: nervous system; P: anti-parasitic products; R: respiratory system; S: sensory organs; and V: several others

The distribution of prediction results across different therapeutic property labels of drugs is illustrated in Fig. 9. The distribution of labels for the gold standard and for the predictions is almost same. However, we predicted more targets for drugs with the label “J” (anti-infectives for systemic use). This result suggests that drugs with that therapeutic property label have more potential to target proteins, and should be studied further for broader use. Furthermore, we examined the association between known targets and predicted targets for each drug. We found that 111 out of 623 drugs known targets and predicted targets are neighbors in the protein-protein interaction (PPI) network based on the BioGRID database [19]. Notably, for the drug Flavopiridol hydrochloride (CID: 5687969), investigated for use/treatment in leukemia (lymphoid), the predicted target HSP90AA1 (Entrez ID: 3320) is a neighbor to eight known targets in the PPI network (Entrez IDs: 983, 1017, 1019, 1020, 1021, 1022, 1025, 1956). Recent research has shown the gene HSP90AA1 is related to hematological malignancies [20].

## Discussion

The ability of DTI prediction is essential and have improved substantially in recent years, but a paucity of data and lack of efficient algorithms leads to hardly systematic DTI prediction. Currently, two advances are poised to facilitate new strategies. First, the LINCS project, launched in 2010, is able to provide rich transcriptome data. Second, deep learning methods have been applied successfully in biomedical research. Nonetheless, even deep neural network has a strong ability of automatically extracted high-level features, the performance of the network is related with the depth and breadth of the layers, and the risk of over-fitting increases resulting from too complicated architecture. Especially after the dimensionality of genome-wide expression declines to 978, how to design an effective architecture of deep neural network for further learning features remains a challenge, and it is difficult to explain such abstract representations.

In order to make the model have better potential for DTI prediction, we rebuilt the objective function for decreasing the PoPC, but validation accuracy went down as well. Because whether a new DTI was reliable depended on the distance score. In future work, we will explore a probability to find an inside property for evaluating new DTI potential.

## Conclusion

In this work, we proposed a framework for DTI prediction based on transcriptome data in the L1000 database gathered from drug perturbation and gene knockout trials. The pipeline of our framework included a combination of data from drugs and genes, as well as negative data sampling. As a result of the increasing availability of data and GPU computing, the DNN employed in our framework served as an effective tool for feature extraction and classification. Once the DNN was trained, the results demonstrated that our framework can discovery more reliable DTIs than found by other methods. Furthermore, this conclusion was validated across platforms with a high percentage of overlap interactions. These findings also demonstrated that our model can integrate transcriptome data from drugs and genes, and has wider prospects for predicting DTIs and improving the drug discovery process.

## Abbreviations

DNN: Deep neural network
DTIs: Drug-target interactions
GBDT: Gradient boosting decision tree
LINCS: Library of integrated network-based cellular signatures
LR: Linear regression
NIH: National institute of health
PoPC: Percentage of positive cases
PPI: Protein-protein interaction
R&D: Research and development
RF: Random forest
VC: Voting classifier

## References

[1] Lounkine E, Keiser MJ, Whitebread S, Mikhailov D, Hamon J, Jenkins JL, Lavan P, Weber E, Doak AK, Côté S (2012). Large scale prediction and testing of drug activity on side-effect targets. Nature.

[2] Dudley JT, Deshpande T, Butte AJ (2011). Exploiting drug–disease relationships for computational drug repositioning. Brief Bioinform.

[3] Law V, Knox C, Djoumbou Y, Jewison T, Guo AC, Liu Y, Maciejewski A, Arndt D, Wilson M, Neveu V (2014). Drugbank 4.0: shedding new light on drug metabolism. Nucleic Acids Res.

[4] Günther S, Kuhn M, Dunkel M, Campillos M, Senger C, Petsalaki E, Ahmed J, Urdiales EG, Gewiess A, Jensen LJ (2008). Supertarget and matador: resources for exploring drug-target relationships. Nucleic Acids Res.

[5] Davis AP, Murphy CG, Johnson R, Lay JM, Lennon-Hopkins K, Saraceni-Richards C, Sciaky D, King BL, Rosenstein MC, Wiegers TC (2012). The comparative toxicogenomics database: update 2013. Nucleic Acids Res.

[6] Campillos M, Kuhn M, Gavin AC, Jensen LJ, Bork P (2008). Drug target identification using side-effect similarity. Science.

[7] Bleakley K, Yamanishi Y (2009). Supervised prediction of drug–target interactions using bipartite local models. Bioinformatics.

[8] Wang Y, Zeng J (2013). Predicting drug-target interactions using restricted boltzmann machines. Bioinformatics.

[9] Yamanishi Y, Araki MA, Honda W, Kanehisa M (2008). Prediction of drug-target interaction networks from the integration of chemical and genomic spaces. Bioinformatics.

[10] Yamanishi Y, Kotera M, Kanehisa M, Goto S (2010). Drug-target interaction prediction from chemical, genomic and pharmacological data in an integrated framework. Bioinformatics.

[11] Xia Z, Wu LY, Zhou X, Wong ST (2010). Semi-supervised drug-protein interaction prediction from heterogeneous biological spaces. BMC Syst Biol.

[12] Chen Y, Li Y, Narayan R, Subramanian A, Xie Xs (2016). Gene expression inference with deep learning. Bioinformatics.

[13] Wagner AH, Coffman AC, Ainscough BJ, Spies NC, Skidmore ZL, Campbell KM, Krysiak K, Deng P, Mcmichael JF, Eldred JM (2016). Dgidb 2.0: mining clinically relevant drug–gene interactions. Nucleic Acids Res.

[14] Kuhn M, Szklarczyk D, Franceschini A, Von MC, Jensen LJ, Bork P (2012). Stitch 3: zooming in on protein-chemical interactions. Nucleic Acids Res.

[15] Zhang M, Li H (2009). Tree kernel-based SVM with structured syntactic knowledge for BTG-based phrase reordering. Proceedings of the 2009 Conference on Empirical Methods in Natural Language Processing: Volume 2 - Volume 2, EMNLP ’09.

[16] Kutner MH, Nachtsheim CJ, Neter J. Applied Linear Regression Models (5th Ed.)Technometrics. 2004; 26(4).

[17] Prinzie A, Van den Poel D (2008). Random forests for multiclass classification: Random multinomial logit. Expert Syst Appl.

[18] Srivastava N, Hinton G, Krizhevsky A, Sutskever I, Salakhutdinov R (2014). Dropout: a simple way to prevent neural networks from overfitting. J Mach Learn Res.

[19] Chatr-Aryamontri A, Breitkreutz BJ, Oughtred R, Boucher L, Heinicke S, Chen D, Stark C, Breitkreutz A, Kolas N, O’Donnell L (2017). The biogrid interaction database: 2015 update. Nucleic Acids Res.

[20] Kliková K, Pilchova I, Stefanikova A, Hatok J, Dobrota D, Racay P (2016). The role of heat shock proteins in leukemia. Klinicka Onkol Cas Ceske Slovenske Onkologicke Spolecnosti.

